# An Automatic Framework for Nasal Esthetic Assessment by ResNet Convolutional Neural Network

**DOI:** 10.1007/s10278-024-00973-7

**Published:** 2024-01-29

**Authors:** Maryam Ashoori, Reza A. Zoroofi, Mohammad Sadeghi

**Affiliations:** 1 Control and Intelligent Processing Center of Excellence, School of Electrical and Computer Engineering, College of Engineering, University of Tehran, Tehran, Iran; 2 Tehran University of Medical Sciences, Imam Khomeini Hospital Complex, Tehran, Iran

**Keywords:** Nasal base, Symmetry, Landmarks, Automatic framework, Hybrid model, Combined model

## Abstract

Nasal base aesthetics is an interesting and challenging issue that attracts the attention of researchers in recent years. With that insight, in this study, we propose a novel automatic framework (AF) for evaluating the nasal base which can be useful to improve the symmetry in rhinoplasty and reconstruction. The introduced AF includes a hybrid model for nasal base landmarks recognition and a combined model for predicting nasal base symmetry. The proposed state-of-the-art nasal base landmark detection model is trained on the nasal base images for comprehensive qualitative and quantitative assessments. Then, the deep convolutional neural networks (CNN) and multi-layer perceptron neural network (MLP) models are integrated by concatenating their last hidden layer to evaluate the nasal base symmetry based on geometry features and tiled images of the nasal base. This study explores the concept of data augmentation by applying the methods motivated via commonly used image augmentation techniques. According to the experimental findings, the results of the AF are closely related to the otolaryngologists’ ratings and are useful for preoperative planning, intraoperative decision-making, and postoperative assessment. Furthermore, the visualization indicates that the proposed AF is capable of predicting the nasal base symmetry and capturing asymmetry areas to facilitate semantic predictions. The codes are accessible at https://github.com/AshooriMaryam/Nasal-Aesthetic-Assessment-Deep-learning.

## Introduction

The nose is a vital element of a person’s esthetic appearance of the face that affects the overall appearance, esthetics, and attractiveness of the facial [1, 2]. The appearance and balance of the nose as important features of rhinoplasty deeply affected by the nasal base [3]. The nasal base so-called alar-columellar complex provides an ideal starting point to develop an analytic approach to quantitative analysis of nasal shape [4] that can be useful to distinguish the face characteristics (for example [5]). This area of nose is a common source of patient dissatisfaction and neglecting of it would lead to some revision rhinoplasty [6]. Some of the complications in rhinoplasty that significantly affect facial attractiveness [2] are asymmetry and deformation appearance [7]. Symmetry can be considered a major factor in nasal base esthetics [8] especially, by using selfies in the digital age, the rate of requests for a more symmetrical nose is increased [9, 10].

Recent developments in artificial intelligence, machine learning, and deep learning techniques have opened new avenues for efficient knowledge discovery from healthcare data [11] which can be trained to carry out tasks that are either challenging or time-consuming for surgeons [12, 13]. Deep learning algorithms are a subset of machine learning algorithms that have led to the construction of several novel deep neural network architectures that are able to illuminate patterns and features that are not always visible to the human eye [11].

Several researchers studied rhinoplasty based on computer technology such as introducing simulation or prediction models for the nasal shape that esthetically matches the patient’s face [14, 15], constructing three-dimensional (3D) facial images from two-dimensional images, and producing 3D simulation models to revolutionalize the practice of functional and esthetic rhinoplasty [16, 17]; the others are listed in [18]. Also, A parametric model (PM) is used to describe objectively nasal base shape [4], and a classification system is created by evaluating and comparing the PM with the categorization by surgeons [19]. Machine learning was used to simulate rhinoplasty results according to the criteria of the doctors surveyed [13]. Finally, deep learning was used to predict rhinoplasty status accurately [20] and patient’s age before and after rhinoplasty [21] and to find which geometric facial features that affect attractiveness in order to considered them within the rhinoplasty procedures [22].

In the latest years, deep learning-based object detection algorithms have played an important role in reducing human efforts in the processing of modern approaches. Object detection algorithms based on deep learning such as region-based convolutional neural networks (R-CNN) [23], Fast R-CNN [24], and Faster R-CNN [25] are characterized by the bounding boxes and categories probabilities for each object. Faster RCNN as a deep object detection algorithm utilizes region proposal networks (RPNs) to generate image regions that provide better performance and more speed for object detection. Object detection to recognize landmarks [26] is one of the most state-of-the-art methods that solves the problem of facial landmark detection [27, 28]. In the most recent research works of the latest years [29], two-stage object detector methods have excellent performance in object recognition and localization accuracy [30]. The advantages of the R-CNN family as a two-stage method, rather than the one-stage detectors are as follows: (1) utilizing the sampling heuristics to deal with class imbalance; (2) regressing the object box parameters by two-step cascade; (3) describing the objects according to two-stage features [31]. However, the above discussed previous works related to the nasal base have some limitations that addressed as follows:

1. In the previous works of the researchers, the symmetry of the nasal base was considered only for evaluating the results of rhinoplasty on cleft palate patients [32–36].
2. In these works, extracting the nasal base landmarks has been taken by manual methods using either direct or indirect anthropometry [32, 35–37]. The important key is the lack of an accurate and rapid automated method to detect the landmarks of the nasal base.
3. In other related research, the symmetry of the nasal base has been studied using some quantitative methods to survey the geometry features that are limited to the statistical methods [4, 8, 32, 34, 37–39]. Barnes and et. Al [4] utilized the lateral deviation (symmetry) of the nasal base as a parameter of a polar function without calculating the value of the symmetry. [8] Presented a clinical technique to improve the symmetry of the columella and nostrils. Then used the ${\chi }^{2}$ test to compare the results of pre and post-operative based on patients’ opinion [34]. Applied descriptive statistics to compare the nasal symmetry of infant with unilateral cleft lip with or without cleft palate between time points from frontal, lateral, and submental views [37]. Utilized Student’s *t* test to analyze narsi symmetry of the patients were treated by using the Hotz plate. [38] used analysis of variance and equality of two proportions tests to compare the symmetry after fat grafting in paranasal and midface groups based on manual extracted measurements and evaluator rating. In [32], Pietruski et al. conducted a validation study to develop a computer system as a tool for objective anthropometric analysis of the nasolabial region. In addition,the practical application of the system was further confirmed through a comparative objective analysis of nasolabial morphology and symmetry in the both healthy individuals and the cleft subjects [39]. It is important to note that in the both of the last works the specified number of landmarks was set by the user. However, we can’t find deep learning algorithms for analyzing the nasal base symmetry. Moreover, it is crucial to highlight that none of the abovementioned studies included a scoring system for evaluating the symmetry of the nasal base.

Therefore, until now there is no unique AF which able to evaluate the symmetry of the nasal base based on deep learning algorithms. So, it is important to propose an exact AF to assess the symmetry value of nasal base before and after rhinoplasty. Also, this paper pays attention to adopting the Faster R-CNN technique to detect the nasal base inside the image and then recognize the nasal base landmarks (Appendix 1). The main motivation of this research is to suggest an AF that is compatible with human opinion to reduce the role of human factors to evaluate the symmetry of the nasal base. The remainder of the paper is as follows:The “Materials and Methods” section illustrates the materials and methods; the “Results and Discussion” section describes the experimental results. Finally, the paper is concluded in the “Conclusions” section.

## Materials and Methods

**Fig. 1:**
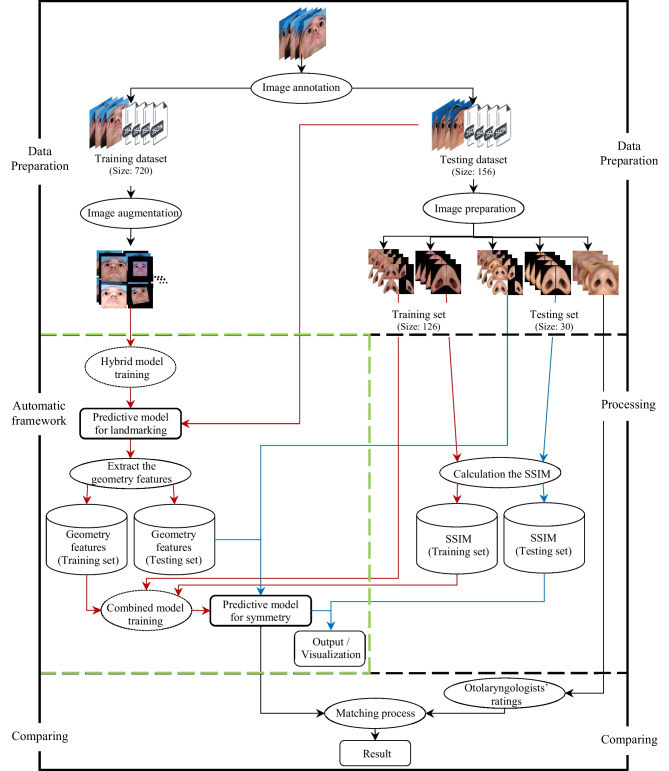
Detailed block diagram of the proposed method to predict the symmetry of the nasal base. The red, blue, and black arrows correspond to the training, testing, and other phases, respectively. The enclosed area between the green dashed lines shows the AF

Figure 1 shows the block diagram of the proposed method which involves three main steps: data preparation, processing, and comparison. In the initial step, the septorhinolpasty dataset (SRD) consisting of the preoperative and one-year postoperative photographs of 438 primary rhinoplasty patients (comprising 740 women and 136 men, totaling 876 image data) was randomly divided into a training dataset (80%, 720 images) and a testing dataset (20%, 156 images). Image annotation was applied to all images in the SRD. Subsequently, image augmentation (25 augmented images per input image) was performed on each image in the training dataset, and the nasal base region was cropped based on the columellar axis. Additionally, tiled image versions of the testing dataset were generated. In the second step, the augmented annotated training dataset was used to train the hybrid model for nasal base landmarks recognition. The original images of the testing dataset (156 images) were fed to the predictive model for landmarking during the testing phase to extract the geometry features. The tiled images and geometry features of the training set (126 images) were utilized as input, and structural similarity index measure (SSIM) [40] was used as the output for the combined model during the training phase. The predictive model for symmetry utilized the testing set (30 images) in conjunction with geometry features to predict the symmetry value of the nasal base. Finally, in the last step, the matching process was investigated to compare the results of the predictive model for symmetry and otolaryngologists’ ratings.

### Dataset Description and Preparation

The color profile photographs of 438 primary rhinoplasty patients (370 women and 68 men) are selected from the database of all patients who had been referred to the otolaryngology office of the third author from 2010 to 2019, where almost all of these patients were elective for cosmetic procedures. While their initial motivation for seeking treatment was cosmetic enhancement, in a substantial portion of subjects (potentially around half), concurrent medical treatment was also provided alongside the cosmetic procedure. The mean age and standard deviation of the patients at the time of surgery was 30.17 ± 5.01 years (range 13–75 years). The skin of the subjects included the types II, III, and IV of skin tone categories in the Fitzpatrick scale [41].

Preoperative and 1-year postoperative photographs were taken from frontal, lateral, and basal views with a Canon 60 D camera in high resolution according to the standard guidelines [42] for clinical photography. The resolution of the images was 72 dpi, and photography was conducted without the use of flash due to enough ambient lighting. The image quality was not compromised by refraining from applying any compression during the image acquisition. As the surgery was septorhinoplasty and there was sensitivity, structured illumination was used to capture images of the patient. The photographs were taken in specialized studios approved by the surgeon and were not color-calibrated. Additionally, if the imaging conditions are not met, the surgeon repeats the image. All patient’s photographs were included in the study after giving written informed consent. Full ethical approval was granted by the University of Tehran research ethics committee. The preparation process of the SRD for training the deep learning models includes collecting data, data cleaning, and format conversion, bounding box annotation and labeling, dataset partition, and data augmentation.

Furthermore, in order to assess the robustness of the hybrid model, we construct a new multi-ethnicity rhinoplasty dataset (MERD) [43, 44] containing paired facial images of 100 rhinoplasty patients of different races and nations extracted from publicly available websites. The MERD includes categories of Ethnic Rhinoplasty, Middle Eastern Rhinoplasty, Latino Rhinoplasty, Asian rhinoplasty, and African American Rhinoplasty. The demographic composition of the MERD consists of 74 women and 26 men (with ages approximately ranging from 18 to 70 + years). The selection criteria for images from these websites included (1) relevance to the scope of this research, (2) a minimum image resolution equal to that of the SRD, and (3) the availability of six views of the face (frontal, lateral (right and left), three-quarter oblique (right and left), and basal). We gathered images depicting frontal, lateral, three-quarter oblique, and basal views of rhinoplasty subjects, in accordance with the available categories of the rhinoplasty images on the websites. Subsequently, we manually annotated them with 18 nasal base landmarks. In terms of size and resolution, the images of SRD and MERD have been resized to equal size, but the resolutions of MERD images were either 72 or 96 dpi.

### Proposed Automatic Framework

#### The Proposed Hybrid Model

**Fig. 2:**
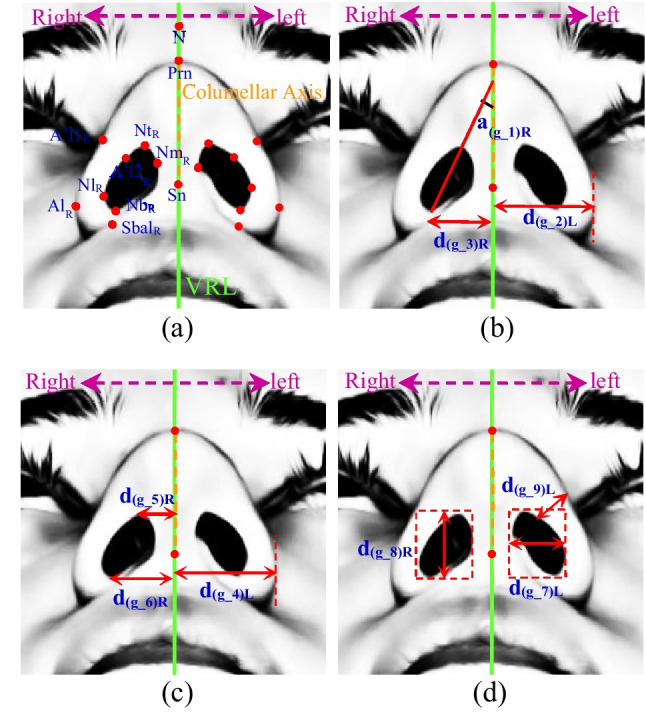
Landmarks (**a**) and geometry features (**b**)-(**d**) of the nasal base. The vertical reference line (VRL) corresponding to the facial midline was set as a perpendicular line crossing Nasion, Subnasale, and Gnathion:

In this subsection, we designed a novel hybrid model combining different architectures including a mixture of Faster R-CNN and CNN models to detect nasal base and to predict nasal base landmarks coordinates automatically. Firstly, the augmented data which was obtained from the training dataset (80% for training: 508 women and 72 men and 20% for validation: 114 women and 26 men) was used to train the hybrid model. Labelme [45, 46] is used to annotate objects, and json file is created for each image to comprise nasal base landmark annotations. There are several landmarks of the nasal base in surgical books (Appendix 1) that produce geometry features including nine scale values $\mathcal{F}=[{f}_{g\_1}, {f}_{g\_2},{f}_{g\_3},{f}_{g\_4},{f}_{g\_5},{f}_{g\_6},{f}_{g\_7},{f}_{g\_8},{f}_{g\_9}]$ that are calculated via Eqs. (1–2) and are shown in Fig. 2.

1 $${f}_{g\_1}={a}_{\left(g\_1\right)L}/{a}_{\left(g\_1\right)R}$$

2 $${f}_{g\_i}={d}_{\left(g\_i\right)L}/{d}_{\left(g\_i\right)R} i=2, 3,\dots , 8,9$$

where ${f}_{g\_1}$ represents the angular ratio of the angulation of the long axis of the left nostril (${a}_{\left(g\_1\right)L}$) and the angulation of the long axis of the right nostril (${a}_{\left(g\_1\right)R}$) and ${f}_{g\_i}$ represents the i-th geometry ratio feature, ${d}_{\left(g\_i\right)L}. {d}_{\left(g\_i\right)R}$ are the i-th distances of left and right sides of nasal base, left/right midalar widths ($i=2$), horizontal distance between the left/right subalare and the facial midline ($i=3$), horizontal distance between the left/right alare and the columellar axis ($i=4$), left/right midcolumellar apex width ($i=5$), left/right midcolumellar base width ($i=6$), left/right nostril width ($i=7$), left/right nostril height ($i=8$), and left/right thickness of ala ($i=9$), respectively.

**Fig. 3:**
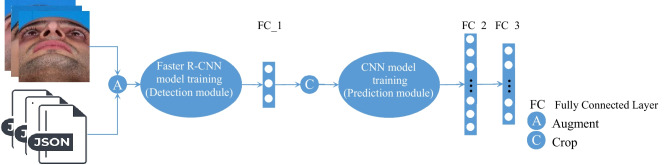
The hybrid model combined the Faster R-CNN and CNN models

We use Faster R-CNN with VGG16 as the backbone network for the object detection task to generate a bounding box around the nasal base inside the input image (detection module). Then, the input is cropped based on this bounding box to have a cropped nasal base. After that, we set again the landmark annotations based on the corresponding bounding region coordinates to build a predictive model that takes a set of cropped nasal base boxes and object annotations as input. Then a customized pre-trained ResNet152V2 model is designed and trained to predict the nasal base landmarks coordinates (prediction module) (Fig. 3). In this method, the landmark predictive model is trained on the bounding box region of the input image, and it can predict the coordinates of landmarks more accurately rather than using the whole image for prediction.

##### Implementation Details

In the detection module, the ground-truth box of the nasal base, which is obtained according to the pronasal and alars coordinates of object annotations, is used to determine the bounding box region in the images. It is trained using Adam optimizer [47] with a learning rate of 1e-5. We selected the small initial learning rate since the pre-trained VGG16 model is not proper for intense changes. In the prediction module, we utilized the Adam optimizer and “LearningRateScheduler” callback with initalpha equal to 1e-3 to calculate the learning rate depending on the current training epoch. The activation function of all convolution layers is the LeakyReLU [48] function. The value of the batch size of the prediction module is 32.

##### Data Augmentation

Image augmentation techniques are used to enhance the efficiency and result of the model. The flipping, rotating, and color transformation data augmentation was used to improve the quality and generalization of images [49] so the hybrid model is trained by random augmentations: color jitter, flipping, shifting, rotating, and scaling. The number of generated augmented samples from each data sample is 25.

##### Training Loss

The task of detecting the object contains both classification (object recognition) and regression (localization of the object) learning problems. The loss function of Faster R-CNN as a two-stage detector can be unified as in (3):

3 $$\mathcal{L}\left(\left\{{p}_{i}\right\},\left\{{t}_{i}\right\}\right)=\frac{1}{{N}_{cls}}\sum_{i}{\mathcal{L}}_{cls}\left({p}_{i},{p}_{i}^{*}\right) +\lambda \frac{1}{{N}_{reg}}\sum_{i}{p}_{i}^{*}{\mathcal{L}}_{reg }\left({t}_{i},{t}_{i}^{*}\right)$$

4 $${\mathcal{L}}_{cls}\left({p}_{i},{p}_{i}^{*}\right)=-{\text{log}}\left[{p}_{i}^{*}{p}_{i}+\left(1-{p}_{i}^{*}\right)\left(1-{p}_{i}\right)\right]$$

5 $$\begin{aligned}{\mathcal{L}}_{reg }\left({t}_{i},{t}_{i}^{*}\right)&={smooth}_{L1}\left({t}_{i}-{t}_{i}^{*}\right)\\&=\left\{\begin{array}{ll}0.5{\left({t}_{i}-{t}_{i}^{*}\right)}^{2}&if \left|{t}_{i}-{t}_{i}^{*}\right|<1\\ \left|{t}_{i}-{t}_{i}^{*}\right|-0.5&otherwise \end{array}\right.\end{aligned}$$

where $i$ is the index of an anchor in a mini‐batch, ${p}_{i}$ is the predicted probability of the anchor $i$ being an object, ${p}_{i}^{*}$ is the ground-truth label that is equal to one if the anchor is positive, and is zero if the anchor is negative. The vectors ${t}_{i}$ and ${t}_{i}^{*}$ represent 4 parameter coordinates of the predicted box and ground-truth box associated with a positive anchor, respectively, and λ is the weight balance parameter also, and ${N}_{cls}$ and ${N}_{reg}$ are the mini-batch sizes and the number of anchor locations, correspondingly. The smooth function calculates the loss function between the ground-truth and the predicted box. We formulate the task of predicting the nasal base landmark coordinates as a regression problem. The loss function of the model is the mean absolute error (MAE).

##### Evaluation Metrics

Since in a given image, the aim of object detection is to find out where objects are located and each object belongs to which category; therefore, the well-known metrics, such as precision and recall, are not sufficient for this task. To effectively evaluate the performance of the detection module, the precision (P), the recall rate (R), F1 score (F1), and the mean average precision (mAP) are selected to evaluate the detection ability of the model. The metrics of MAE and the normalized mean errors (NME) [50] are used to measure the performance of the prediction module are given as follows:

6 $$NME=\frac{1}{n}\sum_{j}^{n}\left(\frac{1}{{d}_{j}}\sum_{k}^{m}{\Vert {\widetilde{Y}}_{j}\left(:,k\right)-{Y}_{j}\left(:, k\right)\Vert }_{2}\right)$$

where $n$ and $m$ are the numbers of the samples (images) and landmarks, respectively, ${Y}_{j}$ is the ground-truth landmarks, ${\widetilde{Y}}_{j}$ is the corresponding estimated landmarks, ${Y}_{j}\left(:. k\right)$ is the kth column of ${Y}_{j}$, and ${d}_{j}$ is $\sqrt{width * height }$ that is the square root of the ground-truth bounding box.

In constructing the hybrid model, various experiments are performed to fine-tune the architecture and the learning process, including changing the pre-trained model, batch size, normalization scheme, learning rate, and the number of augmentation and layers. It should be noted that we examined several schedules of “LearningRateScheduler” as the standard, linear, step-based, polynomial learning rate and exponential decay for training the prediction module then, the best result of them is selected as the result of the models.

#### Combined Model Based on the Geometry Features and Tiled Images

**Fig. 4:**
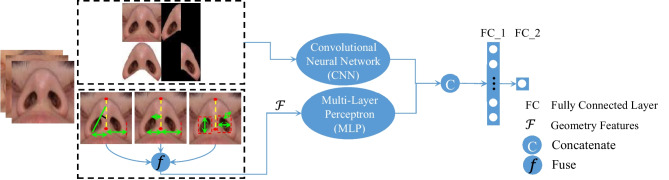
The combined model (CNN and MLP) to predict the nasal base symmetry

After constructing the hybrid model, the testing part of the data (156 images) is partitioned into two parts: the training set (126 images) for the symmetry predicting model and the testing set (30 images). The 156 accumulated nasal base images included 118 women and 38 men patients. To prepare the images, first, the goal area of the nasal base is cropped from the original image after standardization (resizing the images to a uniform size for annotation of the pupils). Additionally, the rotation of the patient’s head is removed to ensure that the inferior right pupil connects to the inferior left pupil, which should be parallel to a line of the Frankfort plane. Then, the image of the nasal base is masked with white color to separate the nasal base region of the image from the background. Typically, white color background is more efficient for masking irrelevant parts of the image [51] and id used to extract the region of interest (ROI). After that, the nasal base is divided into two regions by the line connecting pronasale and subnasale Fig. 4. Finally, a tiled image is constructed according to a vector of length 4 (the total number of tiles in the tiled image). The tiled normalized version of images and geometry features ($\mathcal{F}$) of the training set (80% for training and 20% for validation) is utilized to train the network to find the regression model. The tiled image is passed through the CNN, and the symmetry value is used as the target predicted value. This approach permits the CNN to learn from all images rather than trying to pass the images one at a time, it also enables the CNN to learn discriminative filters from all images at once. The *z*-score method is used to obtain the normalized pixel value (NPV) as in (7):

7 $$NPV\left(x,y,z\right)=\frac{PV\left(x,y,z\right)-\mu (PV\left(z\right))}{\sigma (PV\left(z\right))}$$

where $PV\left(x.y.z\right)$ is the pixel value of the coordinate (*x*,*y*) and channel (*z*). $\mu (PV\left(z\right))$ and $\sigma (PV\left(z\right))$ are the mean and standard deviation values of pixels in channel *z* of the training set. Also, the geometry features are transformed into a common range [0,1]; then, the min–max normalization method is used to calculate the normalized geometry feature (${NGF}_{i}$) for each geometry feature $i$ by (8):

8 $${NGF}_{i}=\frac{{GF}_{i}-{GF}_{min}}{{GF}_{max}-{GF}_{{\text{min}}}}$$

Here, ${GF}_{max}$ and ${GF}_{min}$ are the maximum and minimum values of the $i$-th geometry feature ($\mathcal{F}$), respectively. MLP is trained with geometry features, and CNN is trained with tiled images; then, CNN and MLP are combined to estimate nasal base symmetry. Then the obtained ground-truth symmetry by SSIM was used as a combined model target. The combined model was compiled using Adam optimizer with the learning rate value set at 1e-3 and batch size equal to 8. The loss function and performance metric of the combined model are MAE and the root mean square error (RMSE), respectively. Also, the Pearson correlation coefficient (PC) is used to measure the performance of the model. Several experiments were examined to fine-tune hyperparameter settings to reach optimal performance. The final architecture follows the combination of MLP and CNN models depicted in Fig. 4. The detailed description of the structure of the parameter setup of the combined model components is given in Appendix 2.

### Comparing the Outcomes

In this stage, the results generated by the automatic framework and the otolaryngologists’ ratings were analyzed. The testing set (30 images) was given to otolaryngologists (2 men and 2 women) through a visual questionnaire and asked them to rate the perceived symmetry (completely symmetric, very symmetric, slightly symmetric, asymmetric, and completely asymmetric). It should be noted that all the images are assigned to the patient without any defect in the nasolabial region.

## Results and Discussion

In this section, the performance of the proposed symmetry AF is evaluated. Firstly, the efficiency of the introduced hybrid model is explained; then, the performance of the combined models is analyzed. The transfer learning technique is applied in the hybrid model based on the CNN approach such that Faster R-CNN with pre-trained VGG16 is used to extract bounding box information and a deep learning model with ResNet152V2 as the backbone is exploited to detect landmarks coordinates. Then, the deep CNN and multi-layer perceptron neural network models are integrated by concatenating their last hidden layer to evaluate the nasal base symmetry based on geometry features and tiled images of the nasal base. Finally, the process of matching is executed between the AF results and otolaryngologists’ ratings. All experiments were performed on a Windows machine pre-installed with a 64-bit Win 10 Pro. It has a GTX 1080 Ti 22 GB GPU, 32 GB of RAM, and an Intel(R) Xeon(R) CPU E5-2699 v4 @ 2.20 GHz. Also, the AF is developed by using the Python programming language.

### Evaluating the Hybrid Model

**Fig. 5:**
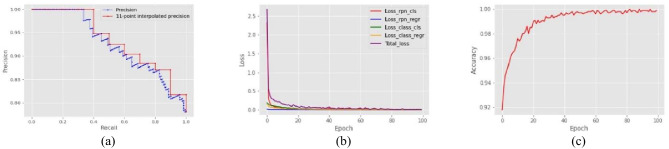
PR curve (precision at 11 recall levels) (**a**), training and validation loss for the Faster RCNN (**b**), training and validation accuracy for the Faster RCNN (**c**)

We obtained the results of the precision rate, recall rate, F1 score, and mAP value of the “nasal_base” class for the Faster RCNN (Table 1). These reported values of the results are achieved according to the testing dataset (156 images). In addition, the plots of the PR curve, loss, and accuracy metric plots are shown to further evaluate the efficiency of the Faster R-CNN (Fig. 5) such that the minimum total loss of 0.0085 and maximum accuracy of 0.9996 acquired during the process of model training in epoch 96. Besides, we applied the Fast R-CNN with VGG16 for the nasal base detection and the performance-based comparisons in the object detection methods indicate that the Faster R-CNN model outperforms the Fast R-CNN model (Table 1). The paired *t*-test analysis with a 0.05 significant level was used to compare the performance metrics of Faster R-CNN and Fast R-CNN. The findings revealed a significant difference in the precision rate and mAP metrics as indicated by the last row of Table 1.

**Table 1:** Performance of the detection module for the Faster and the Fast R-CNN methods

	Precision rate	Recall rate	F1 score	mAP
Faster R-CNN	0.78	1	0.88	0.92
Fast R-CNN	0.69	1	0.81	0.87
*P*-value	**0.00**	0.40	0.88	**0.01**

**Fig. 6:**
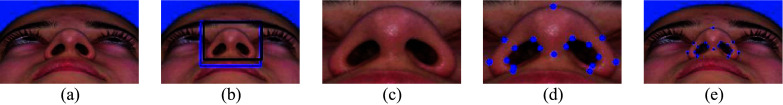
Qualitative result of the hybrid model. Input image (**a**), the output of the detection module (**b**), cropped region (**c**), output of the prediction module (**d**), and the returned landmarks on the input image (**e**). The black and blue bounding boxes are detected and ground-truth bounding boxes (**b**)

The prediction module is trained to detect the nasal base landmarks based on the predicted bounding box extracted by the Faster R-CNN model. The network weights of the module were updated after improving the validation loss from previous epochs. At the end of the training, we pick the model that gives the best performance of validation loss in terms of MAE and NME to evaluate the validation and test datasets and to use later for the landmark predicting process. Table 2 demonstrates the MAE, NME, RMSE, PC, Mean, and Std of them for the prediction module on the SRD. The validation set is used to tune the parameters of the regressor, and the test set is used only to assess the performance of the fully specified regression model. Evaluating the validation and the test sets using the trained prediction module shows that MAE, NME, and RMSE are low for the two sets. The independent *t*-test was conducted at a significance level of 0.05 to compare the performance metrics of the validation and test sets. The results indicated no significant difference among the four metrics of the two sets, suggesting that the prediction module is equally effective at nasal base landmarking in both the validation and test sets. Figure 6 shows the qualitative results of the hybrid model.

**Table 2:** The performance metrics (also their corresponding values of the mean and Std in all epoch) of alignment results of the nasal base

Partition	MAE	Mean (Std)	NME	Mean (Std)	RMSE	Mean (Std)	PC	Mean (Std)
Validation	0.0442	0.0542 (0.0536)	0.0162	0.0201 (0.0170)	0.1210	0.1245 (0.0607)	0.8607	0.8490 (0.0730)
Test	0.0359	0.0673 (0.0849)	0.0137	0.0268 (0.0359)	0.0953	0.0965 (0.1291)	0.9011	0.7181 (0.5238)
*P*-value	**0.4079**		**0.4347**		**0.4341**		**0.3801**

It should be pointed out that we examined two pre-trained VGG-16 and Resnet50 architectures for the detection module and utilized Resnet152V2 and Xception backbones for the prediction module. The hybrid model integrates both detection and prediction techniques in order to provide accurate predictions because each technique is responsible for a different task. Also, the hybrid model decreases the human role in landmark detection which facilitates using it.

### Performance of the Combined Model

The combined model consisted of the CNN and MLP trained to estimate the nasal base symmetry via the tiled images and the geometry features. To do this, we randomly selected 126 images of the testing dataset (156 images) for training the combined model. The weights of the network were set to update only when the validation loss function improved from previous epochs. After training for 100 epochs, we roll back to the model with the lowest validation loss in terms of the MAE based on the training dataset. The model performance metrics (MAE, RMSE, PC) for the validation set (31 images) are 0.1363, 0.0665, and 0.7669, and the values of these metrics for the test set (30 images) are 0.1585, 0.0863, and 0.8394, respectively.

**Fig. 7:**
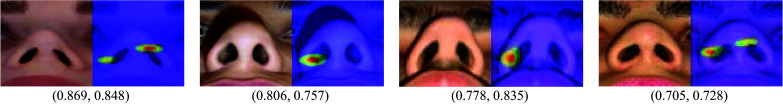
The original image and the heatmap visualization of the cropped nasal base region. The pair values show (the ground-truth value, the predicted values by AF)

In this experiment, we examined the AF to measure the prediction results of the nasal base symmetry. Some sample images of the testing set and their heatmap visualization with the symmetry predicted results of the nasal base images by AF beside ground-truth (or the SSIM value) are shown in Fig. 7. The heatmap is generated through an intersection of the heatmaps derived from both the CNN and the geometric features. The CNN heatmap highlights the tiles used by the network as important features in tiled images, while the geometric feature heatmap is generated from features with a large distance. The resulting heatmap identifies the region of the nasal base that disrupts symmetry.

Therefore, after data preparation, the clinical application of AF is according to this process that, when a new nasal base image comes in, the landmarks are detected by using the hybrid model. Then, based on the detected landmarks, the nine paired geometry features are calculated. Then the derived geometry features and the tiled image are fed to the combined model. Finally, the predicted symmetry value obtained from the AF and the heatmap visualization of the nasal base is shown to users.

### Matching Process of the AF and Otolaryngologists’ Results

The matching process of the results of the AF for the testing set (30 images) and otolaryngologists’ ratings reports that in 19 cases of 30 patients, the combined model predicts the symmetry value according to the opinions of specialist doctors. The results of the matching process showed that the exact and fine matchings of AF with the otolaryngologists’ ratings are 0.6333 and 0.7666. The considered range for transforming quantitative ranges to qualitative ranks is based on the otolaryngologists’ opinions. The important factors that cause gaps between the otolaryngologists’ ratings and the results of the combined model are as follows: there are no standard angles of images, errors in human vision, and errors in the combined model.

### Ablation Study

In order to investigate the effectiveness of the detection module, an extensive ablation study was conducted with the goal of surveying the impact of the detection module to decrease the error of the prediction module’s output. The trained prediction module, after 100 epochs, is evaluated by MAE, the mean average pixel error (MAPE) [52] (9), RMSE, and PC metrics to compute the landmark estimation errors.

9 $$MAPE= \frac{1}{n}\sum_{j}^{n}\sum_{k}^{m}{\Vert {\widetilde{Y}}_{j}\left(:,k\right)-{Y}_{j}\left(:, k\right)\Vert }_{2}$$

#### Module Robustness Analysis

The proposed AF uses the principle of object detection before landmarks recognition. Extensive experiments on augmented data are conducted. To assess the efficacy of the detection module, two models are formed: Model I, prediction module without detection module based on the whole image and Model II, prediction module with detection module based on the cropped nasal base region. Note that Model I and Model II are trained only with the SRD training dataset (Fig. 1). Assessing the outputs of Model I and Model II is done in two methods: the output of Model I is transformed to the scale of the output of Model II (Algorithm 1) and on the contrary (Algorithm 2).

**Algorithm 1:**
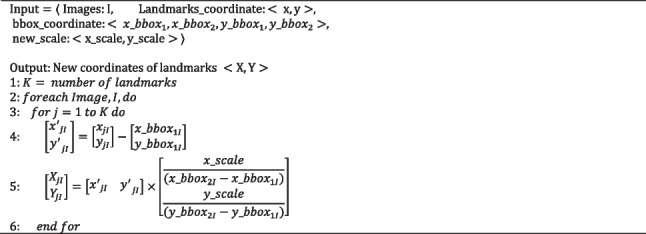
Transformation to zoom-in for the nasal base region (maximize)

**Algorithm 2:**
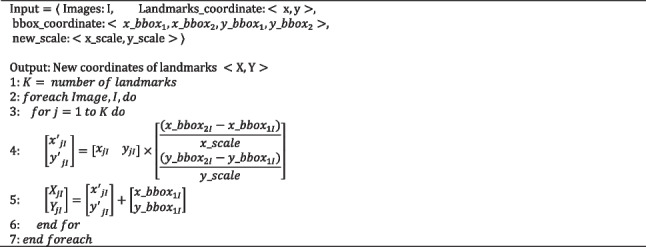
Transformation to zoom-out for the nasal base region (minimize)

**Fig. 8:**
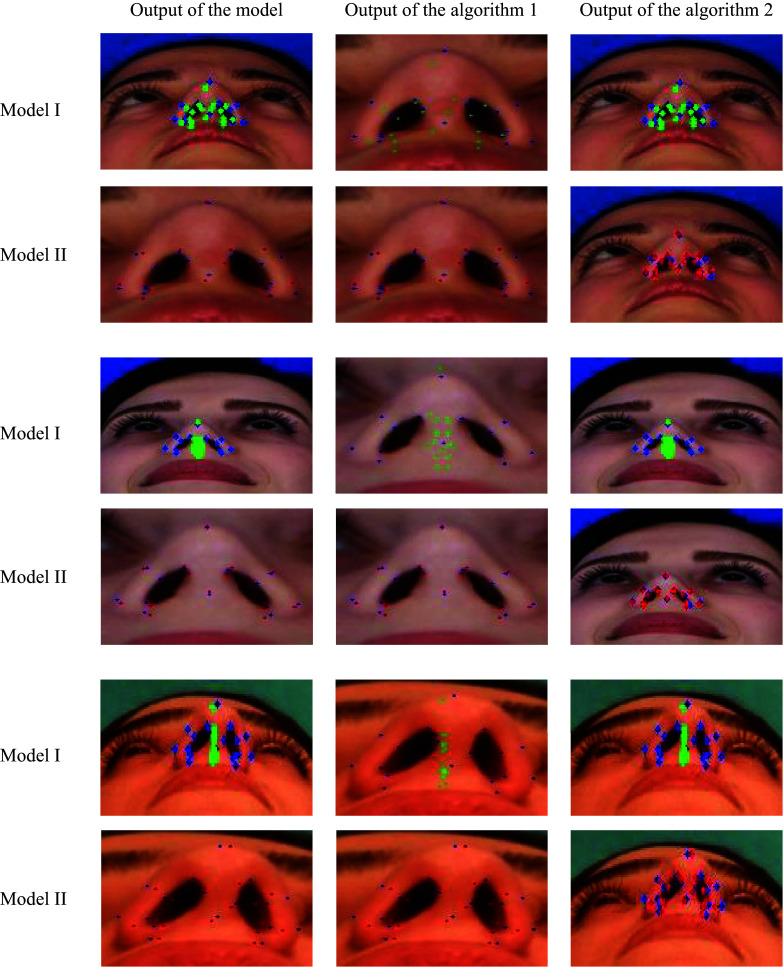
Qualitative results of the AF on SRD. Blue: ground-truth landmarks. Green: detected landmarks by Model I. Red: detected landmarks by Model II. The first column is the model output, the second column is the results of zoom-in using algorithm 1, and the third column is the results of zoom-out by algorithm 2

To compare the performance of the two models, the paired *t*-test at a significance level of 0.05 is implemented. The *P*-values of Table 3 show that there is a significant difference between the evaluation metrics (MAE, MAPE, RMSE, and PC) of the Model I and Model II for both datasets. Since the values of the MAE, MAPE, and RMSE of the Model II are less or equal to the Model I, Model II has a better performance, independent of algorithms 1 and 2. This means that the learning on the cropped region of the image can be more effective for image feature extraction (Fig. 8). It should be noted that the MAE, MAPE, and RMSE values are higher on MERD, which indicates the error of the models in testing these images. This error can be attributed to the diverse nature of the images and, at some times, the lack of training of the model on various types of nasal bases.

**Table 3:** Performance of the prediction module for Model I and Model II

Dataset	Transformation method	Model	MAE	MAPE	RMSE	PC
SRD	Algorithm 1	I	0.0375	0.2870	0.0478	0.9447
		II	0.0329	0.2577	0.0429	0.9529
		P-value	**0.0000**	**0.0000**	**0.0000**	**0.0002**
	Algorithm 2	I	0.0077	0.0604	0.0101	0.9763
		II	0.0079	0.0604	0.0101	0.9817
		P-value	**0.0004**	**0.0021**	**0.0021**	**0.1401**
MERD	Algorithm 1	I	0.0796	0.6404	0.1067	0.7632
		II	0.0533	0.4313	0.0719	0.8813
		P-value	**0.0000**	**0.0000**	**0.0000**	**0.0004**
	Algorithm 2	I	0.0257	0.2150	0.0358	0.8135
		II	0.0140	0.1154	0.0192	0.9262
		P-value	**0.0000**	**0.0000**	**0.0000**	**0.0000**

#### The Impact of Landmarks

A study was conducted to analyze the impact of incorrect landmarks on the performance of the prediction module. This involved randomly shifting the position of correct 3–7 landmarks on two datasets by 1–5 pixel points in different directions (top, bottom, right, and left). The results of paired *t*-tests, as shown in Table 4, indicate that there is a significant difference between the Model ${\text{I}}$ and Model $\widehat{{\text{I}}}$, except for the algorithms 2 for MERD dataset. However, no significant difference was observed between Model ${\text{II}}$ and Model $\widehat{{\text{II}}}$, suggesting that Model ${\text{II}}$ is more robust in the presence of incorrect landmarks compared to Model ${\text{I}}$. Additionally, the *P*-value between Model $\widehat{{\text{I}}}$ and Model $\widehat{{\text{II}}}$ declares a significant difference in the evaluation metrics (MAE, MAPE, and RMSE) for both datasets. Furthermore, the model evaluation metrics (except PC) with incorrect landmarks are slightly greater than those with correct landmarks (PC: 0.9169 vs. 0.9565, 0.9479 vs. 0.9817, 0.7669 vs. 0.8813, 0. 8632 vs. 0.9262).

**Table 4:** Impact of landmarks on the prediction module for two datasets according to Model I and Model II ( ^ represents the model with the incorrect landmarks. *P*-value^*⁎*^ is between $\widehat{I}$ and $\widehat{II}$ models)

Dataset	Transformation method	Model	MAE	*P*-value	MAPE	*P*-value	RMSE	*P*-value	PC	*P*-value
SRD	Algorithm 1	$\widehat{{\text{I}}}$	0.0464	0.0048	0.3583	0.0109	0.0597	0.0109	0.9165	0.1670
		I	0.0375		0.2870		0.0478		0.9447	
		$\widehat{{\text{II}}}$	0.0323	0.8530	0.2652	0.7850	0.0442	0.7850	0.9169	0.0486
		II	0.0329		0.2577		0.0429		0.9529	
		*P*-value^⁎^	**0.0083**		**0.0499**		**0.0499**		**0.9992**	
	Algorithm 2	$\widehat{{\text{I}}}$	0.0094	0.0181	0.0740	0.0389	0.0123	0.0389	0.9525	0.1304
		I	0.0077		0.0604		0.0101		0.9763	
		$\widehat{{\text{II}}}$	0.0080	0.9879	0.0671	0.5357	0.0112	0.5357	0.9479	0.0806
		II	0.0079		0.0604		0.0101		0.9817	
		*P*-value^⁎^	**0.0000**		**0.0000**		**0.0000**		**0.3296**	
MERD	Algorithm 1	$\widehat{{\text{I}}}$	0.0944	0.0000	0.7332	0.0000	0.1222	0.0000	0.7511	0.2822
		I	0.0796		0.6404		0.1067		0.7632	
		$\widehat{{\text{II}}}$	0.0717	0.6054	0.6011	0.9168	0.1002	0.9168	0.7669	0.0000
		II	0.0533		0.4313		0.0719		0.8813	
		*P*-value^⁎^	**0.0000**		**0.0000**		**0.0000**		**0.0004**	
	Algorithm 2	$\widehat{{\text{I}}}$	0.0274	0.2915	0.2311	0.2958	0.0385	0.2958	0.7719	0.1180
		I	0.0257		0.2150		0.0358		0.8135	
		$\widehat{{\text{II}}}$	0.0198	0.0554	0.1693	0.0631	0.0282	0.0631	0.8632	0.1053
		II	0.0140		0.1154		0.0192		0.9262	
		*P*-value^⁎^	**0.0003**		**0.0014**		**0.0014**		**0.0020**	

#### The Impact of Noise in Images

We utilized Gaussian noise, as observed in recent studies on assessing robustness [53–56], to study its impact on the images. Subsequently, we compared the outcomes of the noise dataset with those of the non-noise dataset (Table 5). The results of the paired *t*-test to investigate the effect of noise on the Model I and Model II indicate a significant difference between the noisy ($\ddot{{\text{I}}}$) and noiseless (I) modes, while for the Model II, there is no significant difference between the noise ($\ddot{{\text{II}}}$) and noiseless (II) modes. Therefore, the robustness of Model II, which is based on object detection, is confirmed. Moreover, the P-value comparison between Model $\ddot{{\text{I}}}$ and Model $\ddot{{\text{II}}}$ suggests a significant disparity in the evaluation metrics (MAE, MAPE, and RMSE) for both datasets.

**Table 5:** Performance of the noise and non-noise on two datasets applying Model I and Model II ($\ddot{I}$ and $\ddot{II}$ represent the models with the noise. *P*-value^*⁎*^ is between $\ddot{I}$ and $\ddot{II}$ models)

Dataset	Transformation method	Model	MAE	*P*-value	MAPE	*P*-value	RMSE	*P*-value	PC	*P*-value
SRD	Algorithm 1	$\ddot{{\text{I}}}$	0.0579	0.0006	0.4651	0.0143	0.0775	0.0143	0.8382	0.0001
		I	0.0375		0.2870		0.0478		0.9447	
		$\ddot{{\text{II}}}$	0.0428	0.7120	0.3535	0.5982	0.0589	0.5892	0.8703	0.1283
		II	0.0329		0.2577		0.0429		0.9529	
		*P*-value	**0.0101**		**0.0289**		**0.0289**		**0.2755**	
	Algorithm 2	$\ddot{{\text{I}}}$	0.0122	0.0000	0.1001	0.0000	0.0167	0.0000	0.8872	0.0617
		I	0.0077		0.0604		0.0101		0.9763	
		$\ddot{{\text{II}}}$	0.0105	0.0393	0.0889	0.2320	0.0148	0.2320	0.9032	0.0312
		II	0.0079		0.0604		0.0101		0.9817	
		*P*-value	**0.0000**		**0.0003**		**0.0003**		**0.3187**	
MERD	Algorithm 1	$\ddot{{\text{I}}}$	0.1739	0.0313	1.4912	0.0005	0.2485	0.0005	0.1641	0.0000
		I	0.0796		0.6404		0.1067		0.7632	
		$\ddot{{\text{II}}}$	0.0822	0.5719	0.7035	0.1740	0.1172	0.1740	0.6868	0.0000
		II	0.0533		0.4313		0.0719		0.8813	
		*P*-value	**0.0383**		**0.0019**		**0.0019**		**0.0069**	
	Algorithm 2	$\ddot{{\text{I}}}$	0.0486	0.0000	0.4273	0.0000	0.0712	0.0000	0.4980	0.0000
		I	0.0257		0.2150		0.0358		0.8135	
		$\ddot{{\text{II}}}$	0.0229	0.0777	0.1993	0.0640	0.0332	0.0640	0.8053	0.2870
		II	0.0140		0.1154		0.0192		0.9262	
		*P*-value	**0.0000**		**0.0000**		**0.0000**		**0.0000**	

The limitations of prior studies include the use of statistical analysis to examine medical interventions in surgery [8, 34, 38], reliance on user participation until the result is obtained [4, 32, 37], and the inability of the presented methods to provide a quantitative measure of symmetry [4, 32, 37, 39]. These studies have advantages in addressing the issues of a specific range of patients and offering computer-based approaches. In our research, we have addressed the limitations of previous studies by employing an automated method with repeatability and high accuracy to predict the quantitative value of symmetry.

The issue of imprecise bounding box localization presents a significant area for future research. In an attempt to minimize this problem in this research, after accurate annotations for training through manual and labor-intensive labeling by motivation of [57], we initiated with a larger bounding box. However, our team has thoroughly investigated this topic and plans to address it in an upcoming article, utilizing insights from recent publications [58–60]. As this matter exceeds the scope of the current work, we have conducted a comprehensive examination and analysis to be incorporated into the forthcoming article. The authors have openly shared the model, including its code, and have made it available to all interested researchers, while excluding the dataset.

## Conclusions

This study presented a novel automatic framework (AF) based on deep learning algorithms for evaluating nasal base symmetry. The goal of this framework is to improve the previous symmetry methods based on quantitative and qualitative assessments. The suggested AF is compatible with otolaryngologists’ ratings and reduces the role of human factors in evaluating symmetry, thereby aiding surgeons in planning expedited operations and improving surgical outcomes. In the proposed framework, two detection and prediction modules are combined into an integrated hybrid model to address the problem of nasal base landmark recognition. The standard data augmentation techniques and Adam optimizer are implemented to achieve the optimum value of the loss function during the training process. The evaluated results of the trained prediction module show that learning on the cropped region of the image can be more effective for image feature extraction. A combined model was developed and trained using tiled images and the geometric features of the nasal base to predict nasal base symmetry. The results of the matching process confirmed that the AF was consistent at 0.7666 with the otolaryngologists’ ratings which demonstrates the efficiency of the proposed framework. Also, the capability of the proposed AF can be seen in decreasing the rhinoplasty reconstructive surgery, especially, in cleft palate subjects that require a precise description of the symmetry and a visual representation of its changes. To sum up, the proposed AF is capable of sensing the nasal base symmetry and capturing the asymmetry regions to demonstrate a heatmap visualization for patients and otolaryngologists. Consequently, our future research will concentrate on studying and designing unsupervised learning-based object detectors to develop our hybrid model and construct a real-time symmetry AF. Furthermore, we suggest that future studies of the introduced hybrid model can be utilized in various application domains.

## Appendix 1

List of landmarks of the nasal base [32, 61]

**Table:**

Facial landmark	Abbreviation	Description
Alare _L/R_	Al _L/R_	The most lateral point in the curved of each ala
Subalare _L/R_	Sbal _L/R_	The point at the lower limit of each alar base, where the alar base disappears into the skin of the upper lip
Al _L/R_	Al _L/R_	The marking level at the midportion of the alae
Nostril tip _L/R_	Nt _L/R_	The most cranial point of the inner border of the nostril
Nostril base _L/R_	Nb _L/R_	The most caudal point of the inner border of the nostril
Nostril mediale _L/R_	Nm _L/R_	The most medial point of the inner border of the nostril
Nostril laterale _L/R_	Nl _L/R_	The most lateral point of the inner border of the nostril
Pronasale	Prn	The most protruding point on nasal tip
Subnasale	Sn	The midline point at the junction of the inferior margin of the columella and the upper lip skin

## Appendix 2

General parameters of the structure utilized to setup the combined model.

**Table:**

		Layers	Input size	Kernel size	Strides	Activation function	Padding	Output size
MLP		Dense_1	9	-	-	ReLU	-	18
		Dense_2	18	-	-	ReLU	-	9
CNN	Block_1	Conv2D_1	64 × 64 × 3	3 × 3 × 32	1,1	-	same	64 × 64 × 32
		BatchNormalization_1	64 × 64 × 32	-	-	-	-	64 × 64 × 32
		Activation_1	64 × 64 × 32	-	-	ReLU	-	64 × 64 × 32
		MaxPooling2D_1	64 × 64 × 32	2 × 2	-	-	-	32 × 32 × 32
	Block_2	Conv2D_2	32 × 32 × 32	3 × 3 × 64	1,1	-	same	32 × 32 × 64
		BatchNormalization_2	32 × 32 × 64	-	-	-	-	32 × 32 × 64
		Activation_2	32 × 32 × 64	-	-	ReLU	-	32 × 32 × 64
		MaxPooling2D_2	32 × 32 × 64	2 × 2	-	-	-	16 × 16 × 64
	Block_3	Conv2D_3	16 × 16 × 64	3 × 3 × 128	1,1	-	same	16 × 16 × 128
		BatchNormalization_3	16 × 16 × 128	-	-	-	-	16 × 16 × 128
		Activation_3	16 × 16 × 128	-	-	ReLU	-	16 × 16 × 128
		MaxPooling2D_3	16 × 16 × 128	2 × 2	-	-	-	8 × 8 × 128
	Block_4	Conv2D_4	8 × 8 × 128	3 × 3 × 256	1,1	-	same	8 × 8 × 256
		BatchNormalization_4	8 × 8 × 256	-	-	-	-	8 × 8 × 256
		Activation_4	8 × 8 × 256	-	-	ReLU	-	8 × 8 × 256
		MaxPooling2D_4	8 × 8 × 256	2 × 2	-	-	-	4 × 4 × 256
	Block_5	Conv2D_5	4 × 4 × 256	3 × 3 × 512	1,1	-	same	4 × 4 × 512
		BatchNormalization_5	4 × 4 × 512	-	-	-	-	4 × 4 × 512
		Activation_5	4 × 4 × 512	-	-	ReLU	-	4 × 4 × 512
		MaxPooling2D_5	4 × 4 × 512	2 × 2	-	-	-	2 × 2 × 512
		Flatten	2 × 2 × 512	-	-	-	-	2048
		Dense_3	2048	-	-	-	-	36
		Activation_6	36	-	-	ReLU	-	36
		BatchNormalization_6	36	-	-	-	-	36
		**Dropout_1**	**36**	**-**	**-**	**-**	**-**	**36**
		Dense_4	36	-	-	-	-	9
		Activation_7	9	-	-	ReLU	-	9
Combined		**Concatenate_1**	[9]	**-**	**-**	**-**	**-**	**18**
		Dense_1	18	-	-	ReLU	-	9
		Dense_2	9	-	-	Linear	-	1
